# The complete mitochondrial DNA of the tropical oyster *Crassostrea belcheri* from the Cần Giò’ mangrove in Vietnam

**DOI:** 10.1080/23802359.2018.1462126

**Published:** 2018-04-12

**Authors:** Romain Gastineau, Đŭc-Hùng Nguyễn, Claude Lemieux, Monique Turmel, Réjean Tremblay, Văn Duy Nguyễn, Ita Widowati, Andrzej Witkowski, Jean-Luc Mouget

**Affiliations:** a Natural Sciences Research and Educational Center and Palaeoceanology Unit, Faculty of Geosciences, University of Szczecin, Szczecin, Poland;; b Faculty of Natural Sciences Pedagogy, Saigon University, Ho Chi Minh City, Vietnam;; c Département de biochimie, de microbiologie et de bio-informatique, Institut de Biologie Intégrative et des Systèmes, Université Laval, Québec, Canada;; d ISMER, Université du Québec à Rimouski, Rimouski, Canada;; e Faculty of Fisheries and Marine Sciences, Diponegoro University, Semarang, Indonesia;; f Mer-Molécules-Santé (MMS), FR CNRS 3473 IUML, Le Mans Université, Le Mans, France

**Keywords:** *Crassostrea belcheri*, mitogenome, Vietnam, mangrove, oyster

## Abstract

The complete mitochondrial genome of the oyster *Crassostrea belcheri* from the Cần Giò’ mangrove in Vietnam has been sequenced. It consists of a circular DNA molecule of 21020 base pairs (bp), coding for 12 proteins, 20 transfer RNAs, and two ribosomal RNAs. Like the mitogenomes of *Crassostrea iredalei* and *Crassostrea* sp. DB1, it contains a non-coding region and two ORFs. The *C. belcheri* mitogenome provides information that could improve the molecular phylogeny of Asian oysters and be useful to the development of oyster aquaculture in South East Asia.

The tropical oyster *Crassostrea belcheri* is cultured commercially in South-East Asia (Tan and Wong 1996; Klinbuga et al. 2000, 2003, 2005) and Indonesian archipelago (Yoo and Ryu 1984). Its commercialization is an important activity in the Cần Giò’ mangrove, located near Hồ Chí Minh City in Vietnam, where it is sold under the vernacular name of Hàu Dép. In this study, we present the complete mitogenome of *C. belcheri*. The specimen was collected in the mangrove in September 2017, in the Lò Vôi river (10°25′55″N; 106°54′530″E), the remaining flesh and shells being conserved at Saigon University. DNA was extracted following a chloroform-isopropanol protocol, and sequencing was provided by the Beijing Genomic Institute, consisting of 4 Gb of 150-bp paired-end reads from fragments of 300 bp, obtained on an Illumina HiSeq 4000. Data were assembled using Ray 2.3.1 (Boisvert et al. 2010) with a k-mer of 31.

The *C. belcheri* mitogenome is 21020 bp long (GenBank accession no. MH051332), making it the third longest mitogenome sequenced among the genus *Crassostrea* (Wu et al. 2010, 2012). The nucleotide composition of this mitogenome is A 28.3%, C 14.3%, G 21.4%, T 36%. It encodes 12 proteins, 20 transfer RNAs, and two ribosomal RNAs. The *rrnL* gene is split into two parts and the *rrnS* gene is duplicated, both features being commonly found among Asian oysters (Wu et al. 2010). As in other *Crassostrea* mitogenomes, there is a major non-coding region whose size is 797 bp in *C. belcheri*. Also, the *C. belcheri* mitogenome shares with *Crassostrea iredalei* (FJ841967) and *Crassostrea* sp. DB1 (JQ060958) two ORFs originating from the duplication of the *NAD2* gene. The latter ORFs are separated from one another by a putative suppressor tRNA gene.

A maximum-likelihood phylogenetic analysis was performed using the complete mitogenome sequences of 11 *Crassostrea* species and the resulting tree was rooted using the mitogenome of *Ostrea edulis (Figure 1)*. *C. belcheri* was recovered between a strongly supported cluster containing *C. hongkongensis*, *C. ariakensis*, *C. nippona*, *C. sikamea*, *C. angulate,* and *C. gigas*, which all have mitogenomes very similar in size and nucleotide composition, and a second robust cluster containing *C. iredalei* and *Crassostrea* sp. DB1, which feature the longest mitogenomes and the two conserved ORFs. The highly supported clade formed by all nine *Crassostrea* species was sister to that containing *C. virginica* and *C. gasar*, both from the American continent.

**Figure 1.**
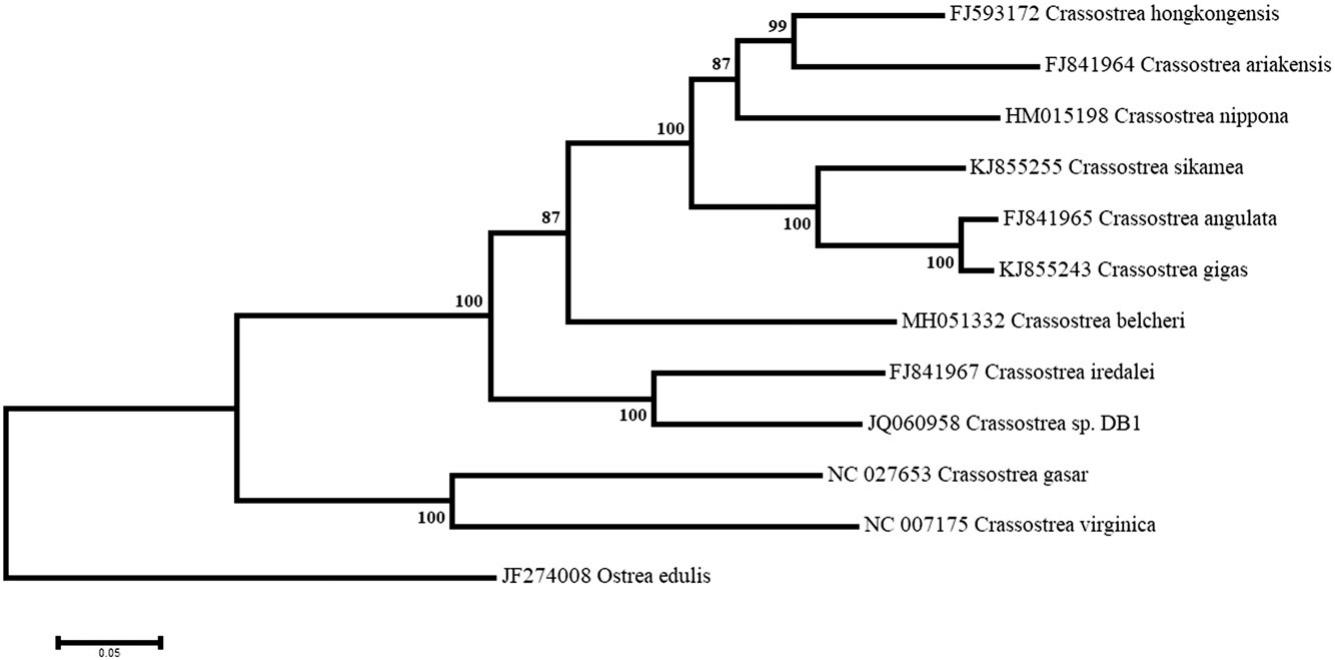
Maximum likelihood phylogeny using the complete mitogenomes of *Crassostrea belcheri* and other species of oysters, with *Ostrea edulis* as an outgroup. The tree with the highest log likelihood (−161490.9077) is shown. Numbers next to nodes are support values obtained after 1000 bootstrap replicates.

The *C. belcheri* mitogenome thus provides new information regarding the phylogeny and genome evolution of Asian *Crassostrea* species. It may also serve as a reliable reference sequence for accurate molecular barcoding of the different populations of *C. belcheri* and identification of specimens, a tool that may be helpful for the development of the aquaculture of this species in South East Asia.
